# Pasteurized autograft reconstruction after resection of periacetabular malignant bone tumours

**DOI:** 10.1186/s12957-016-1065-4

**Published:** 2017-01-09

**Authors:** Xiaoning Guo, Xiaoyang Li, Tang Liu, Cijun Shuai, Qing Zhang

**Affiliations:** 1 Department of Orthopaedics, the Second Xiangya hospital, Central South University, 139 Renmin road, Changsha, 410011 People’s Republic of China; 2 Orthopedic Biomedical Materials Institute, Central South University, Changsha, 410083 People’s Republic of China

**Keywords:** Periacetabular malignant bone tumour, Autograft, Pasteurization, Reconstruction

## Abstract

**Background:**

The treatment of periacetabular malignant bone tumours is challenging. Many methods of reconstruction after internal hemipelvectomy have been reported and each method has its own limitations. The aim of this study was to evaluate the oncological and functional outcomes of pasteurized autograft reconstruction after resection of periacetabular malignant bone tumours.

**Methods:**

Ten patients (six male, four female) with periacetabular malignant tumours, who underwent resection and reconstruction with pasteurized autograft, were retrospectively reviewed. The patients’ average age at diagnosis was 40 years (range 13–65 years). There were five patients with chondrosarcoma, three with osteosarcoma, one with Ewing’s sarcoma, and one with solitary metastatic thyroid carcinoma.

**Results:**

At the last follow-up, seven patients were alive (six with no evidence of the primary disease and one with lung metastasis for 5 months). The three other patients died of metastasis of the primary disease with a mean survival time of 12 (range 8–17) months postoperatively. The mean follow-up time for all patients was 45 (range 8–87) months. Local recurrence rate was 10%. The mean time of bone union was 12 (range 6–21) months after the operation. The mean Musculoskeletal Tumor Society score for all living patients at the last follow-up was 70.5% (range 43.3–86.7%).

**Conclusions:**

Reconstruction with pasteurized autograft is a feasible method for treating periacetabular malignant bone tumours, with satisfactory oncological and functional outcomes and a relatively low incidence of complications.

## Background

With the development in neoadjuvant and adjuvant treatments, surgical techniques, and imaging techniques, the prognosis for malignant bone tumours has considerably improved. However, the treatment of pelvic malignant bone tumours is still challenging because of the anatomical complexity of the pelvis and the difficulty in achieving wide surgical margins and reconstructing large bone and soft tissue defects after tumour resection, especially in the periacetabular region [1, 2].

The methods for the reconstruction of the periacetabular region include (1) arthrodesis [3, 4]; (2) pseudarthrosis [3, 5, 6]; (3) cement reinforced by rods or pins [7, 8]; (4) hemipelvic prosthesis replacement [9–17]; (5) allograft implantation [18–20]; and (6) autograft implantation [8, 18, 21–23].

For autografting, the replacement of the extracorporeally irradiated [21, 22] or autoclaved [8, 18] tumour-bearing bone has been reported by many authors to be a feasible method for reconstruction after pelvic malignant bone tumour resection. To our knowledge, only a few studies have reported reconstruction with pasteurized autograft for periacetabular malignant bone tumours [23, 24].

The purpose of this retrospective study was to evaluate the oncological and functional outcomes of pasteurized autograft reconstruction after resection of periacetabular malignant bone tumours.

## Methods

### Patients

Between February 2007 and August 2012, 10 patients with periacetabular malignant bone tumour underwent tumour resection and replacement with pasteurized autograft in our hospital. Patient characteristics are shown in Table 1. There were six male and four female subjects. The patients’ average age at diagnosis was 40 (range 13–65) years. After preoperative staging, which included plain radiography, magnetic resonance imaging (MRI) of the pelvis, and computed tomography (CT) of the chest, needle biopsy was performed for each patient. There were five patients with chondrosarcoma, three with osteosarcoma, one with Ewing’s sarcoma, and one with solitary metastatic thyroid carcinoma. Seven primary malignant bone tumours were staged as IIB and two were staged as IB according to the Enneking staging system. The solitary metastatic thyroid carcinoma was contained within the innominate bone. In all patients, the pelvic bone structure was not evidently destroyed by the tumour.

**Table 1:** Characteristics and outcomes of patients

Case	Age (years)	Gender	Diagnosis	Enneking stage	Resection	Margin	Follow-up (months)	Oncologic results	Local recurrence	Distant metastasis	Union time (months)	Complications	MSTS (%)
1	27	M	Osteosarcoma	IIB	I + II	M	17	DOD	No	Lung	9	Dislocation	–
2	44	F	Thyroid carcinoma	–	I + II	W	87	NED	No	No	9	Wound necrosis	86.7
3	55	F	Chondrosarcoma	IB	I + II	W	77	NED	No	No	–	Deep infection	43.3
4	19	M	Ewing’s sarcoma	IIB	II + III	C	11	DOD	yes	lung	9		–
5	57	M	Chondrosarcoma	IIB	I + II + III	W	62	NED	No	No	12	Breakage of plate	73.3
6	16	M	Osteosarcoma	IIB	I + II	W	8	DOD	No	Lung	–		–
7	48	F	Chondrosarcoma	IIB	I + II + III	W	54	NED	No	No	12	Wound necrosis superficial infection	73.3
8	65	M	Chondrosarcoma	IIB	I + II	W	47	AWM	No	Lung	21		60.0
9	13	M	Osteosarcoma	IIB	I + II	W	44	NED	No	No	6		80.0
10	53	F	Chondrosarcoma	IB	II + III	M	38	NED	No	No	15		76.6

*W* wide margin, *M* marginal margin, *C* contaminated margin, *MSTS* Musculoskeletal Tumor Society score, *DOD* died of disease, *NED* no evidence of disease of a primary tumour or a primary metastatic disease, *AWM* alive with metastasis of a primary tumour

All patients with osteosarcoma and the patient with Ewing’s sarcoma received neoadjuvant and adjuvant chemotherapy with a high dose of methotrexate, doxorubicin, cisplatin, and isofosfamide. The patient with Ewing’s sarcoma also received adjuvant radiotherapy after local recurrence and lung metastasis. The patient with solitary metastatic thyroid carcinoma received iodine-131 therapy postoperatively.

There were six cases of type I + II resection, two cases of type II + III resection, and two cases of type I + II + III resection according to the Enneking system.

### Surgical technique

A senior surgeon (Qing Zhang) performed all the operations. The operations were performed with the patients in a lateral position maintained by bags and rolls, which allowed semi-supine and semi-prone positions. Whole or part ilioinguinal and Smith-Petersen incisions were used. The external iliac vessels and the femoral and sciatic nerve were carefully dissected and protected. If the anterior superior iliac spine was not invaded by the tumour, the anterior superior iliac spine was osteotomized and retracted inferiorly with the sartorius muscle. If the tumour did not invade the hip joint, the joint capsule was excised. Femoral neck osteotomy was performed with an oscillating saw after the dislocation of the hip. If the hip joint was invaded by the tumour, femoral osteotomy was performed outside the hip joint capsule. The planned levels of pelvic osteotomy were identified. The osteotomies were performed with a Gigli saw. Subsequently, the tumour was completely removed with a layer of normal tissue and the needle biopsy track. If the hip joint was invaded by the tumour, the pelvis and femoral head were removed en bloc. If the tumour did not invade the hip joint, the pelvis and femoral head were removed separately.

**Fig. 1:**
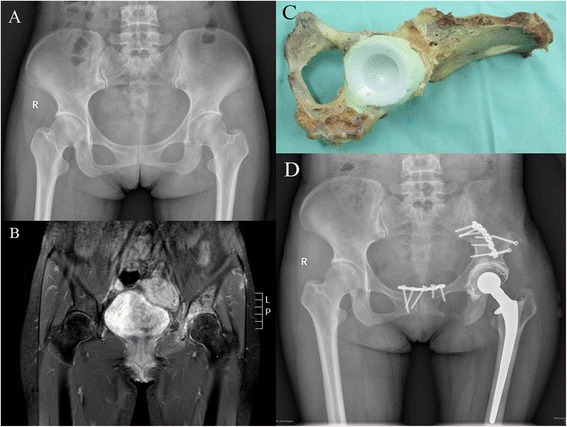
Patient 7 was a 48-year-old female with chondrosarcoma. **a** Preoperative AP radiograph. **b** Preoperative MRI. **c** Pasteurized autograft ready to be returned. **d** AP radiograph 54 months postoperatively. Bone resorption was observed at the inferior ramus of the pubis

The bone was pasteurized as described in our previous reports [25, 26]. Briefly, soft tissue and gross tumour were thoroughly cleared from the specimen. The specimen was then treated in preheated saline at 65 °C for 30 min (YCX-2 Thermostatic Water Bath, Zhengrong Yiqi, China). The acetabulum was reamed extracorporeally according to its original direction until all the cartilage had been removed. Several holes were drilled through the subchondral bone plate of the ilium, ischium, and pubis. The acetabular component was fixed with bone cement in the correct direction (Fig. 1). Areas of defect caused by tumour involvement were filled with bone cement at the same time. The protruding part of the iliac wing which could cause abnormal soft tissue tension was resected. The autograft was returned to its original anatomic site and fixed with plates and screws. Usually, tow reconstruction plates were used, one placed along the arcuate line and pubic ramus and another placed along the posterior side of the ilium and ischial tuberosity. If the anterior superior iliac spine with the sartorius muscle was osteotomized, it was fixed to the autograft with a screw. A standard cemented or cementless femoral component implantation was performed. Thereafter, the muscles were reattached as far as possible to their original position through the drilled holes in the bone to cover the bone and prosthesis with the remaining muscles. The wound was closed with 1 or 2 suction drainages.

### Postoperative treatment

Postoperatively, patients were kept in bed for 2–4 weeks, with the affected extremity in a 15° abduction, 15° hip and knee flexion, and rotary neutral position with an orthosis to prevent dislocation of the hip joint, followed by non-weight-bearing movement. Progressive weight bearing was allowed at postoperative 6 weeks with tow crutches. The drainage tube was removed when the output was <50 ml per day, usually 1–2 weeks after the operation. Intravenous antibiotic was administrated until the drainage tube was removed. Patients started mechanical anti-embolism exercise immediately after the operation. No chemical anti-embolism drug was used.

### Follow-up

All patients were followed up clinically and radiologically (radiograph of the pelvis and chest) every month for 6 months, every 3 months for 2 years, and every 6 months thereafter. A CT scan of the chest was performed every 3 months in the first year and every 6 months subsequently. If there was a suspicion of local recurrence or metastasis, a CT scan was immediately performed. Functional outcome was evaluated using the Musculoskeletal Tumor Society (MSTS) lower extremity score [27] at the final clinical follow-up.

## Results

All patients were followed up either until October 2015 or death. At the final follow-up, seven patients were alive: six with no evidence of the primary disease and one with lung metastasis for 5 months. Three patients (two with osteosarcoma and one with Ewing’s sarcoma) died of primary disease metastasis with a mean survival time of 12 (range 8–17) months postoperatively. The mean follow-up time for all patients was 45 (range 8–87) months, and the mean follow-up time for survived patients was 58 (range 38–87) months.

Resection margins were wide in seven patients, marginal in two patients, and contaminated in one patient. In the current study, if the plane of dissection was 2–3 cm away from the tumour or outside the compartments in all directions, we considered it a wide margin. If the plane did not achieve adequate distance from the tumour in any direction because important vessels or nerves were too close to the tumour, we considered it a marginal margin. If the margin was contaminated by tumour content during the operation, we considered it a contaminated margin. One patient with contaminated margin had local recurrence in soft tissue but not in the bone at 6 months postoperatively, who refused further treatment because lung metastasis was also discovered during this period.

**Fig. 2:**
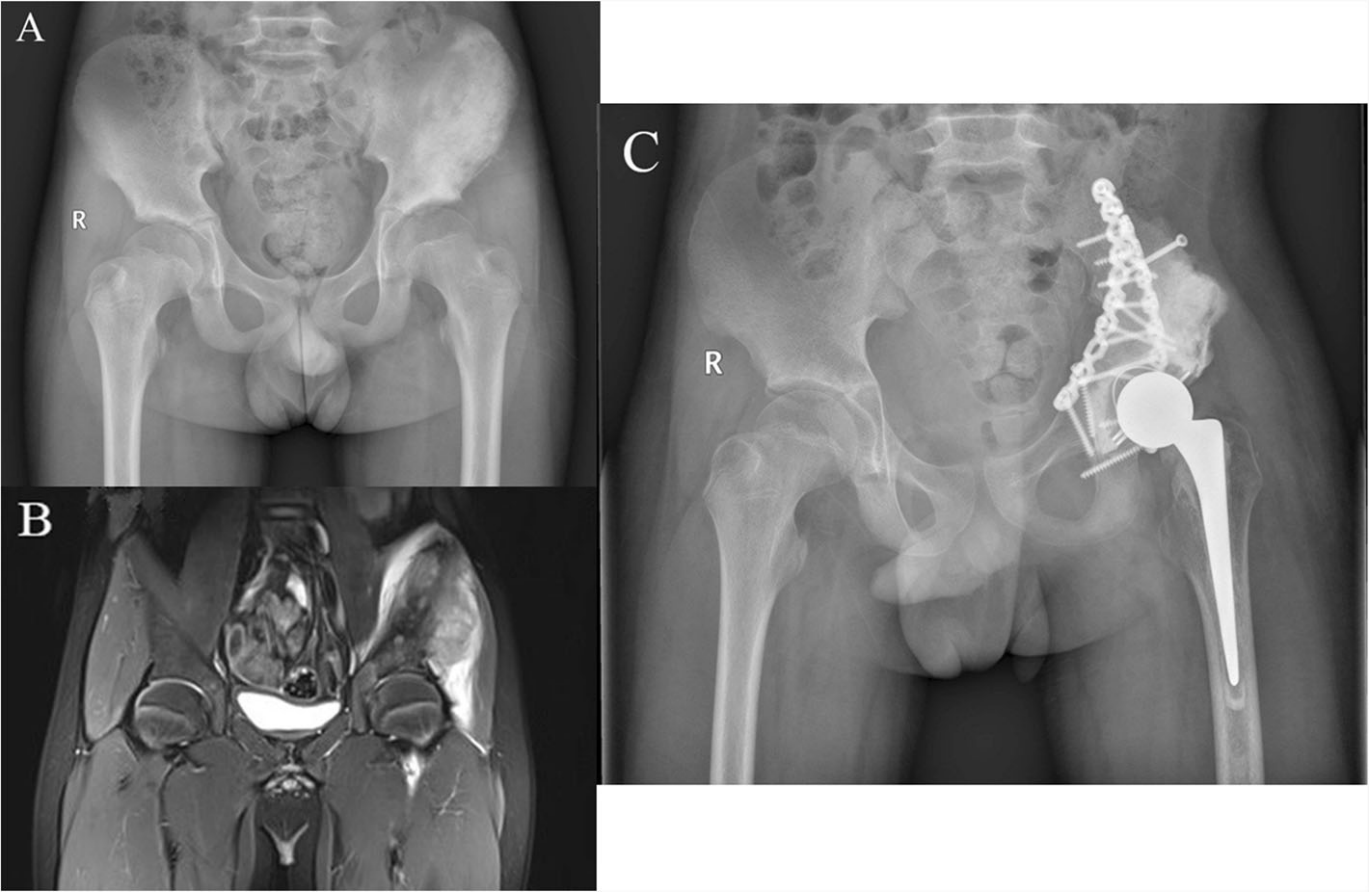
Patient 9 was a 13-year-old male with osteosarcoma. **a** Preoperative AP radiograph. **b** Preoperative MRI. **c** Bone union was achieved at 6 months postoperatively

When a bridging callus appeared between the host bone and pasteurized autograft in an anteroposterior radiograph of the pelvis, we considered that bone union was achieved. Bone union was achieved in eight patients. The mean time of bone union was 12 (range 6–21) months after the operation (Fig. 2). One patient’s autograft was removed because of deep infection before bone union was achieved. Another patient died at 8 months postoperatively because of lung metastasis before bone union was achieved. Bone resorption was found in one patient at the inferior ramus of pubis (Fig. 1). It began at 2 months after the operation and did not progress a year later. No stress fracture or autograft collapse was observed in the current study.

Two patients had wound necrosis; one was treated with change of dressing alone and the other with a superficial infection received debridement. Deep infection occurred in one patient at 4 months postoperatively during the period of adjuvant chemotherapy with isofosfamide. The deep infection was controlled after removing the autograft bone and prosthesis, and no further reconstruction was performed.

A plate which fixed the synchondroses pubis was broken in one patient at postoperative 12 months, but the patient felt no pain at the site and limb function was unaffected. There was no aseptic loosening of prosthesis in the current study.

Dislocation occurred in one patient at 8 days after the operation because of inadequate nursing care. After closed reduction and bed rest for 3 weeks with orthosis, no redislocation occurred. No neurologic complication was observed in the current study.

The mean MSTS score for all survived patients at the final follow-up was 70.5% (range 43.3–86.7%). The mean MSTS score for patients with autograft and prosthesis in situ was 75.0% (range 60.0–86.6%). The MSTS score for the patient whose autograft and prosthesis were removed because of deep infection was 43.3%.

## Discussion

Resection of malignant pelvic bone tumours with a safe margin is difficult. However, internal hemipelvectomy which saves the limb may theoretically achieve the same surgical margin as external hemipelvectomy in most cases [19, 28]. The oncologic outcomes were not significantly different between internal and external hemipelvectomy [1]. The resection of periacetabular bone malignant tumours disrupts force transfers between the trunk and the affected lower limb and affects hip joint function [10]. For reconstruction of the pelvic ring and restoration of hip joint function, various methods are used in clinical practice. Each of these methods has its own limitations.

To obtain adequate hip joint function, the pelvis should be reconstructed as originally as possible to maintain the position of the hip centre of rotation, femur and acetabulum offset, and limb length, to keep an optimal gluteal moment arm [22]. Reconstruction with combined hemipelvic prosthesis could place the acetabulum in the correct position and direction [13]; however, it was difficult to fix the prosthesis in the correct position and direction during the operation, because of the geometrical complexity of the pelvis and large defects in the bone and soft tissue. Computer-aided custom-made hemipelvic prosthesis could help solve this problem [10]. Yet, accurate resection is needed to ensure the correct sitting of the prosthesis; otherwise, the deviation of the acetabular position and direction will occur; thus, the use of a navigation system during resection is recommended [11].

From our point of view, replacement of the treated tumour-bearing bone is relatively easier and an economic way to restore the original position and direction of the acetabulum. Pasteurization is an alternative method to tumour-bearing bone treatment for reconstruction after the resection of a malignant bone tumour [24–26, 29–31]. In the current study, we used pasteurized autografts to reconstruct the pelvis after periacetabular malignant bone tumour resection; the mean MSTS score for patients with autograft and prosthesis in situ was 75.0% (range 60–86.6%) which was comparable to other reports (Table 2). Using an autograft providing acceptable anatomical reconstruction is one of the reasons good hip function was achieved in this study. Apart from the reconstruction of the structure of the pelvis and hip joint, reconstruction of the muscles is also one of the important factors for a good functional outcome [2, 16, 19]. We reattached remain muscles as far as possible to their original position through the drilled holes in the pasteurized bone.

**Table 2:** Comparison with other studies

Author (year)	Number of patients	Method of reconstruction	Mean follow-up duration (months, range)	Overall survival rate (%)	Local recurrence rate (%)	Deep infection rate (%)	Dislocation rate (%)	MSTS (%)
Current study	10	Pasteurized autograft	45 (8–87)	70	10	10	10	70.5
Jeon et al. [23] (2007)	14	Pasteurized autograft	87 (13–142)	78	14	21	36	71.6
Satcher et al. [8] (2003)	15	Autoclaved autograft	56 (12–164)	60	20	0	13	76.6
Wafa et al. [22] (2014)	18	Irradiated autograft	51 (4–185)	50	17	17	6	77.0
Delloye et al. [20] (2007)	24	Allograft	41 (1–137)	33^a^	29	13	8	73.0
Ji et al. [17] (2013)	100	Modular prosthesis	53 (24–102)	64	20	15	9	57.2

*MSTS* Musculoskeletal Tumor Society score

^a^Three deaths were not related to the tumour

Pasteurization of the autograft is sufficient for killing all the tumour cells in the massive bone [29, 30, 32]. Local recurrence rates with pasteurized autograft use were not significantly different compared with prosthesis [26] and allograft [31]. In the current study, one patient (10%) had local recurrence in soft tissue but not in the pasteurized autograft.

There was no effective compartmental barrier for the pelvic tumour [3]. The anatomical structure of the pelvis, the intrapelvic visceral organs, and the neurovascular structures are complex. Although MRI provides an excellent image of the tumour extension, it is difficult to translate this image into a three-dimensional structure during the operation to achieve a safety resection margin [1, 13]. The achieved surgical margin and tumour stage are significant prognostic factors which affect oncological outcome [1]. The overall survival rate in the current study was 70% and the local recurrence rate was 10% which were comparable to other reports (Table 2); however, our mean follow-up duration was only 45 months.

In addition to the primary tumours, the metastatic lesion of the pelvis was one of the indications for en bloc tumour resection and reconstruction in patients with periacetabular malignant tumour. However, the procedure should be limited to patients with solitary metastatic lesion from tumours which have good survival expectancy, such as thyroid, prostate, or breast carcinoma [15, 17, 33]. Nevertheless, some authors do not recommend reconstructing with allograft and recycled tumour-bearing bone for metastatic lesions because of the long rehabilitation period [15]. In this study, one patient with solitary metastatic thyroid carcinoma achieved good functional result (MSTS 86.7%) and was still alive without disease for 87 months at the final follow-up. Although we only presented one patient in the current study, we believe that a solitary metastatic lesion should not be a contraindication for reconstruction with pasteurized autograft for periacetabular malignant tumour because of the relatively long survival expectancy in selected patients.

Reconstruction with a pasteurized autograft is a well-established method in certain countries, particularly in Asia and Africa. It is a simple, easily accessible, and economical alternative to the usual reconstruction modalities with comparable oncological and functional outcomes, in addition to its social and religious acceptance in these countries [23–26, 34, 35]. However, the usage of replacement autograft is limited by the quality of the autograft. The structure of the bone should not be visibly destroyed by the tumour [18, 22, 23]. And using pasteurized autograft would cause the lack of material for the overall histologic assessment of the chemotherapy effect. Moreover, a variety of complications associated with pasteurized autografts have been described, including delayed or nonunion of the junction, graft fracture, collapse, bone absorption, pseudarthrosis, and mechanical implant failure, infection [23–26].

Compared with autoclaving, pasteurization of autograft does not only eliminate malignant cells but also preserves the osteo-inductive activity of the human bone morphogenetic protein [29, 30, 32]. In the current study, if the anterior superior iliac spine was not invaded by tumour, we osteotomized it with the sartorius muscle and fixed it to the pasteurized autograft with a screw. This may have improved the blood supply to the pasteurized autograft for better new bone ingrowth. The mean time of bone union in the current study was 12 months (range 6–21) months postoperatively. It was much shorter than we reported previously; 18 months for the humerus [26] and 19 months for the tibia [25]. There was no nonunion in the current study, while the nonunion rate was 25.0% for the humerus [26] and 54.5% for the tibia [25] in our previous studies. This may have been due to a better blood supply in the pelvis than in the humerus and tibia. The nonunion rate of reconstruction using autograft and allograft after pelvic tumour resection ranged from 0 to 36.8% in other studies [18–23].

There was no case of stress fracture in the current study. Stress fracture rate of the autograft and allograft ranged from 0 to 21.4% in other reports [18–23]. Langlais et al. reported one patient with acetabular fossa stress fracture 2 years after allograft reconstruction. During the revision operation, poorly vascularized bone was found at the fracture site [19]. One reason for the stress fracture occurrence is that dead bone with no self-repair ability was not replaced by the ingrowth of the new bone. The pasteurized autograft could be one of reasons for the absence of stress fracture in the current study because it may have preserved the osteo-inductive activity of the human bone morphogenetic protein. In another study in which pasteurized autografts were used for the reconstruction of periacetabular malignant tumour, the fracture rate was 14% (2/14). The authors assumed that graft disruption caused by the tumour was the reason for the fractures [23]. In the current study, the structures of all autografts were not evidently destroyed by tumour. We also considered this as one of the reasons for the absence of fractures in our study.

Bone absorption is one of the usual complications of pasteurization. Kim et al. reported bone absorption rate was 18% (2/11) in their study, an acetabulum fracture after bone absorption of the pasteurized acetabulum was occurred in one of these patients [24]. In the current study, bone absorption was observed in one patient (10%) at the inferior ramus of pubis 2 months postoperatively, but it did not progress a year later.

There was a breakage of plate which fixed the synchondroses pubis in the current study at 12 months after the operation, but there was no impact on limb function. Nonunion is a significant cause of implant failure. However, there was no nonunion in this patient and we thought that micro motion at the synchondroses pubis was the cause of the breakage. Strong scar healing contributed to the stabilization of the pelvic ring and prevented the patient from experiencing pain and dysfunction. Because there was no impact on limb function, and no pain at the site of breakage, no further treatment was conducted for the breakage of the plate.

Infection is one of the most common and serious complications of reconstruction after resection of pelvic tumours. Deep infection rates range from 0 to 25% and may cause implant removal, amputation, and even death [9, 12–17]. In the current study, one patient (10%) had deep infection 4 months postoperatively, during the period of adjuvant chemotherapy. The deep infection was controlled after the autograft bone and prosthesis were removed. The other patient had a superficial infection which was treated with debridement. Extensive resection, large wound cavity, hematoma, adjuvant chemotherapy, the long duration of surgery, large amounts of blood loss, and the lack of soft tissue coverage are considered reasons for the high infection rate [17, 36–39]. We resected the protruding part of the iliac wing to reduce the tension of the soft tissue and provide better soft tissue coverage as postulated by Langlais et al. [19] and Guo et al. [13]. Muscle flap is recommended for better soft tissue coverage if necessary [14]. Sufficient drainage helps reduce the infection rate by preventing hematoma [17]. We kept the drainage tube in situ, until the output was <50 ml per day. Using antibiotic-laden cement may also help in reducing the infection rate [40].

Dislocation is another common complication in reconstruction after periacetabular tumour resection. Dislocation rates range from 0 to 35.7% and patients with redislocation may need revision [14–17, 23]. One patient (10%) experienced dislocation in this study. Fisher et al. pointed out that the main reason for dislocation was extensive resection of the muscle around the hip joint and recommended ‘buttercup’ exercises for all their patients to prevent dislocation [40]. The faulty positioning of the acetabular cup is also one of the reasons for dislocation. It is difficult to adjust the acetabular cup fixation point when using most hemipelvic prosthesis during operation [10]. Reducing the risk of dislocation might be one of the advantages of reconstruction with pasteurized autograft, because using an autograft restores the original position and direction of the acetabulum.

### Limitations

There were several limitations in the current study. First, the number of patients was small because of the low morbidity of this disease and limited indication for this method. Second, it was a retrospective study without a control group mainly because of the same reason above; thus, we compared our findings with other similar reports. Third, the current study was only a short-term follow-up study; nevertheless, a summary of short-term results is still valuable with regard to early complications and functional outcomes for patients with malignant disease.

## Conclusions

Pasteurized autograft reconstruction is a feasible method for treating periacetabular malignant bone tumours with satisfactory oncological and functional outcome and a relatively low incidence of complications. It has several advantages: (1) provision of adequate anatomical reconstruction for achieve good function, (2) relative ease of fixation during operation, (3) convenient technique for treating autograft, (4) avoidance of disease transmission or immunological reactions, and (5) economic efficiency. However, to avoid graft failure, the structure of the tumour-bearing bone should not be obviously destroyed by tumour, and this may limit the usage of the pasteurized autograft reconstruction to selected patients.

## Abbreviations

CT: Computed tomography
MRI: Magnetic resonance imaging
MSTS: Musculoskeletal Tumor Society Scoring system
